# A Study of Fibroid Vascularization and Vascular Indices With Three-Dimensional Power Doppler and Superb Microvascular Imaging and the Correlation With Heavy Menstrual Bleeding

**DOI:** 10.7759/cureus.71246

**Published:** 2024-10-11

**Authors:** Neeru Malik, Rajiv Ranjan, Rashmi Khatri, Sunita Kumari, Vinayak Malik, Uday K Singh, Aakash Joon, Nikita Madan, Ritu Khatuja, Anupa Singhal

**Affiliations:** 1 Obstetrics and Gynaecology, Dr. Baba Saheb Ambedkar Medical College and Hospital, New Delhi, IND; 2 Radiology, Dr. Baba Saheb Ambedkar Medical College and Hospital, New Delhi, IND; 3 Computer Science, University of Wisconsin–Madison, Madison, USA; 4 Medicine, All India Institute of Medical Sciences, New Delhi, New Delhi, IND; 5 Obstetrics and Gynaecology, ESIC (Employee's State Insurance Corporation) Hospital and Postgraduate Institute of Medical Sciences and Research (PGIMSR) Basaidarapur, New Delhi, IND

**Keywords:** 3d power doppler sonography, heavy menstrual bleeding, leiomyoma, superb microvascular imaging, uterine artery embolization (uae)

## Abstract

Objective

The objective of the study was to evaluate fibroid vascularization and vascular indices with three-dimensional (3D) power Doppler and superb microvascular imaging (SMI) and its correlation with heavy menstrual bleeding (HMB).

Material and methods

A total of 75 women with intramural uterine fibroid: 50 with HMB (Group A) and 25 without HMB (Group B) were recruited for the study. All participants were subjected to transabdominal and transvaginal color Doppler ultrasound. A 3D Power Doppler was used to measure Doppler Indices and flow rate in uterine arteries and the dominant vessel of fibroid. SMI was also used to determine vascularity in the dominant fibroid and to grade the vascularity.

Results

A significant association was observed between Doppler indices in the dominant feeding vessel of fibroid and HMB. Flow rates, namely peak systolic velocity (PSV) and end-diastolic velocity (EDV) were significantly higher. Contrarily, resistance measured as Resistive Index (RI) and Pulsatility Index (PI) was lower in Group A than in Group B. The mean PSV of the dominant feeding vessel in group A was 26.42 ± 4.57 and in group B was 14.56 ± 3.17, with p-value < 0.0001. For EDV, the median value (25th-75th percentile) in women with HMB was 6.76 (6.152-8.74) vs 3.99 (3.2-5.95) in women without HMB (p < 0.0001). A significant association was observed between uterine artery Doppler indices (specifically, PSV and EDV) and HMB. However, no significant association was found between RI and PI of the uterine artery and HMB. In the study of the vascular pattern on SMI, the percentage of women exhibiting both central and peripheral vascularity in Group A and Group B were 72% and 4%, respectively (p < 0.0001). Women in Group A showed a significantly higher proportion of grade 2 and grade 3 vascularity (76% and 20%, respectively), whereas only 4% showed grade 1 vascularity. Conversely, in Group B, all the women showed grade 1 vascularity (p <0.001).

Conclusion

Doppler indices in the dominant feeding vessel of fibroid show a positive correlation with HMB. In the study of the vascular pattern on SMI, a significant proportion of women with HMB exhibited both peripheral and central vascularity and a higher grade. Most women with symptomatic fibroids are of childbearing age and fertility preservation is at times their prime concern. Fibroid vascularization assessed with 3D power Doppler ultrasound and the new advanced SMU tool may prove to be useful in the prediction of response to various non-surgical management options of uterine leiomyoma and thus aid in counseling and appropriate patient selection.

## Introduction

Uterine fibroids are the most common benign tumors of the female reproductive system [1]. The incidence increases with age and is highest by the menopausal age, after which it decreases constantly. By the age of 50, fibroids are prevalent in up to 80% of women [2]. They are benign smooth muscle tumors that are usually asymptomatic but in some cases may seriously impair the patient’s quality of life [3]. The size and location of fibroid is responsible for the symptoms related to it. Most of them being found incidentally during routine gynecological examination, require observation only [4]. Approximately 25-50% of women with fibroids are symptomatic, experiencing heavy menstrual bleeding (HMB), reproductive issues, painful menstruation, pelvic masses associated with pelvic pain, urinary problems, or constipation [5].

The recommended treatment of symptomatic fibroids is myomectomy or hysterectomy, but many women with fibroids avoid surgery because of medical comorbidities or personal preferences. Such patients may be offered minimally invasive uterine-sparing alternatives. The advantages include preserving the uterus and thereby fertility; and reduction in morbidity and recovery time in comparison with hysterectomy. Minimally invasive uterine-sparing procedures include uterine artery embolization (UAE), and magnetic resonance imaging (MRI)-guided high-intensity focused ultrasound (MR-HIFU) [6]. Vascularization plays an important role in these techniques for fibroid treatment and is one of the leading parameters in predicting the outcome of the therapy. Vascularization of fibroid is also directly related to response with medical management like gonadotropin hormone-releasing hormone (GnRH) agonists and letrozole [7]. While UAE has been reported to be more suitable in fibroids with high vascularity [8], MR-HIFU is less suited in these cases [9]. Several previous studies have investigated the changes observed on uterine artery Doppler following UAE, whereas others have reported on the utility of these Doppler changes in predicting treatment outcomes. Assessment of peri-fibroid and intra-fibroid Doppler indices have been found clinically useful in pre-UAE procedures because the overall tumor vascularity is said to be predictive of the therapeutic outcome of UAE [10,11]. Fibroid vascularization (assessed with three-dimensional (3D) Power Doppler ultrasonography (PDUS)) may be a predictor for fibroid-related symptoms, quality of life [12], and uterine fibroid growth [13].

Diagnosis of fibroid is usually made with good accuracy by ultrasound or MRI. Macro-vascularity is assessed by color Doppler ultrasonography (CDUS). Vascular structures that cannot be detected on CDUS, but are evaluated as micro-vessel density (MVD) in pathology preparations, are considered micro-vascularity. A new innovative Doppler ultrasound technique, superb microvascular imaging (SMI), specifically for imaging low flow states, allows visualization of vessels with small caliber and slow velocity [14]. In contrast to conventional Doppler techniques such as PDUS and CDUS, SMI uses a higher frame rate and can detect tiny vessels. PDUS is sensitive and less angle dependent and is a useful modality in case of low blood flow circulation [15]. The 3D Power Doppler vascular indices allow objective assessment of vascularization in the entire volume of the fibroid. 

Very few studies have been conducted to assess the Doppler velocimetry of fibroid vasculature and its correlation with HMB [12,16]. Here, our aim was to study fibroid vascularization and vascular indices (of uterine artery and dominant feeding vessel of fibroid) with 3D PDUS and SMI and its correlation with HMB.

## Materials and methods

This comparative observational study was conducted at the Department of Obstetrics & Gynecology of Dr. Baba Saheb Ambedkar Medical College & Hospital, New Delhi, between June 2023 and February 2024. The study was approved by the Institutional Ethics Committee & Scientific Research Committee, Dr. Baba Saheb Ambedkar Medical College and Hospital (approval number: F.4(60)/2022/BSAH/DNB/PF2118). All participants were informed about the study's purpose and gave their written consent.

Inclusion and exclusion criteria

Patient inclusion criteria in this study were: women with uterine fibroid (already diagnosed on ultrasound) with or without HMV, intramural fibroid, size of fibroid 2-8 cm, age between 20 and 45 years. Patients who had submucosal and sub-serosal fibroids, more than two fibroids of size >2 cm, endometrial hyperplasia, a diagnosis of HMB due to other causes like endometriosis, endometrial carcinoma, adenomyosis, coagulopathy, etc., a recent history of gynecological surgery, active infection or a history of major diseases, and mental illness and cognitive disorder were excluded from the study.

Sample size calculation

Saad et al. observed that RI and PI in women with bleeding were 0.52±0.02 and 0.71±0.05, respectively, and in women without bleeding, it was 0.61±0.09 and 0.93±0.34, respectively [11]. Taking these values as a reference and the sample size ratio as 2:1 (as no. of women with fibroid presenting to outpatient department (OPD) are more in case of HMB), the minimum required sample size with 95% power of study and 5% level of significance was 24 women for controls and 48 women for cases. To reduce the margin of error, the sample size was 75 (50 cases and 25 controls).

Thus, 75 women with uterine fibroid (already diagnosed on ultrasound) with or without HMB were included in the study. They were divided into two groups based on the presence or absence of HMB: Group A included women with HMB (n=50) and Group B included those without HMB (n=25).

Investigations and data collection

All the patients were subjected to a complete history taking which included: the patient’s age, chief complaint and history of present illness, menstrual history (present history of bleeding, including onset, course, duration, amount, and criteria of bleeding pattern), obstetric history, contraceptive history (history of recent use of hormonal contraception), drug history (anticoagulants or antiplatelet drugs), medical history (including chronic hypertension, diabetes, tuberculosis, cancer, blood disease, blood transfusion or any other major medical disorder), surgical history (history of any surgical intervention), personal history, family history, general physical examination including height, weight, and BMI. The presence of pallor, edema, cyanosis, clubbing, icterus, and lymphadenopathy was checked. Thyroid and breast were examined. Temperature, pulse, respiratory rate, and blood pressure were recorded. Abdominal examination was done. A per speculum and bimanual pelvic examination were also performed. Laboratory investigations were done, which included: complete blood count, liver function test, kidney function test, bleeding time, clotting time, and coagulation profile (prothrombin time (PT) and activated partial thromboplastin time (APTT)) in patients who have had HMB since their periods started and had a personal or family history suggesting a coagulation disorder, thyroid hormone test for women presenting with other signs and symptoms of thyroid disease, endometrial biopsy, and Pap smear (Papanicolaou test).

All patients were subjected to transabdominal and transvaginal CDUS, performed by two equally qualified radiologists, using the MyLab™9 Platform (Esaote SpA, Genoa, Italy) ultrasound system with Doppler-enabled convex transducer (frequency of 3.5-5.0 MHz) and transvaginal probe (frequency of 5.0 to 7.5), respectively. The uterus was explored in multiple sections through rotation and tilt. Uterine size, shape, position, internal echo, edge characteristics, endometrial abnormalities, presence of mass, muscle wall thickness, and other information were observed. It was scanned to determine the number of fibroid nodules, identify the dominant/largest nodule, and assess for degenerative changes. The volume of the dominant nodule was calculated from its length, anteroposterior diameter, and transverse diameter using the ellipsoid formula: Length (cm)×Anteroposterior (cm)×Transverse (cm)×0.523.

The 3D Power Doppler was used to measure Doppler indices and flow rate in uterine arteries and the dominant vessel of fibroid. In transvaginal sonography (TVS), the probe is brought in the midline in the longitudinal plane and then again moving laterally; the serpiginous tubular structure is seen at the level of the internal os, the uterine artery. It can also be traced in the transverse axis of the uterus, moving the probe to the level of the internal os, where vessels will be seen on both sides. Doppler velocimetric measurements were also obtained from the peri-fibroid (capsular) and/or intra-fibroid (core) arteries of the dominant fibroid nodule using a Doppler gate sample volume of 1 mm placed in the center of the vessel and a Doppler insonation angle of less than 60°. Dominant fibroid nodule vascularity was determined with SMI as vascular or avascular.

The vascular fibroids were further characterized as having peripheral vascularity only or peripheral and central vascularity. Peripheral vascularity referred to the presence of peri-fibroid (capsular) arteries only, while (peripheral + central) vascularity denoted the presence of both peri-fibroid (capsular) arteries and intra-fibroid (core) arteries simultaneously/concurrently. SMI was also used to grade vascularity according to Adler Grade Classification as grade 0 (absent), grade 1 (minimal), grade 2 (moderate), and grade 3 (marked) [15].

Statistical analysis

Statistical analysis was done using IBM SPSS Statistics for Windows, Version 25.0 (Released 2017; IBM Corp., Armonk, New York, United States). Categorical variables were presented in number and percentage, while continuous variables were presented as mean±SD and median. The normality of data was tested using the Kolmogorov-Smirnov test. If the normality is rejected then a non-parametric test was being used. Quantitative variables were compared using the unpaired t-test/Mann-Whitney test (when the data sets were not normally distributed) between the two groups. Qualitative variables were compared using the Chi-Square test/Fisher’s exact test. A p-value of < 0.05 was considered statistically significant.

## Results

**Figure 1:**
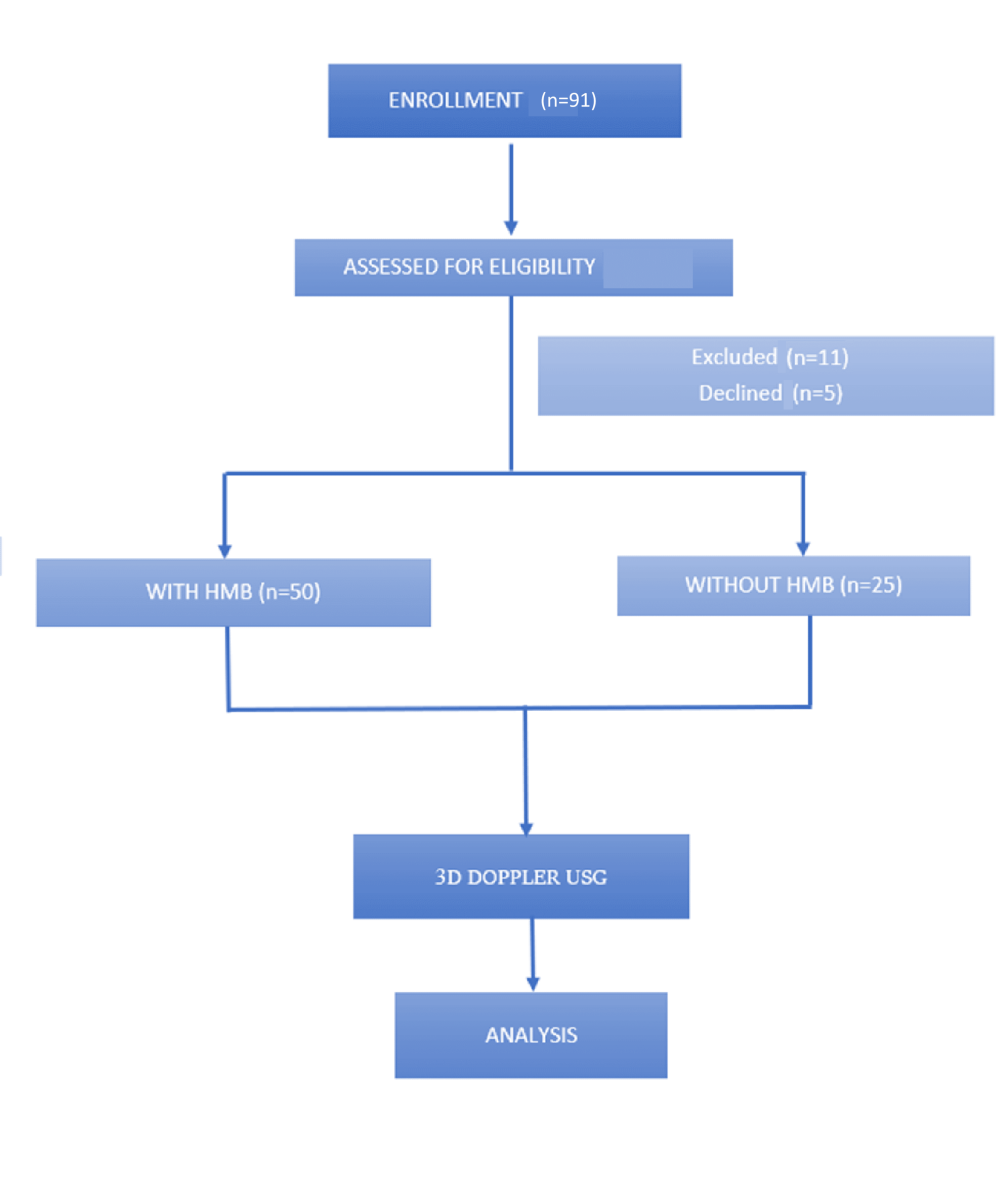
Flow chart of the study HMB: heavy menstrual bleeding; USG: ultrasonography

A total of 91 women with intramural uterine fibroid (pre-diagnosed on ultrasound) were assessed for eligibility out of which 16 women were excluded (11 did not meet the inclusion criteria and five declined to participate). The remaining 75 women were grouped into two groups: Group A (included women with intramural uterine fibroids with HMB) and Group B (included women with intramural uterine fibroids without HMB). Ultrasonography and analysis of both groups were done as described in the previous section. The study process is given in Figure 1.

Table 1 includes the characteristics of the participants. While age, height, and weight show no significant association with HMB, it was observed that those without HMB had significantly higher levels of hemoglobin (Hb) (p-value < 0.0001).

**Table 1:** Characteristics of participants with fibroid uterus with or without heavy menstrual bleeding ‡ Independent t-test, * Fisher's exact test HMB: heavy menstrual bleeding

Characteristics	Participants with HMB (n=50)	Participants without HMB (n=25)	Total (n=75)	P value
Age (years), n (%)				
31 to 35 years	9 (18%)	5 (20%)	14 (18.67%)	0.901^*^
36 to 40 years	20 (40%)	11 (44%)	31 (41.33%)	
41 to 45 years	21 (42%)	9 (36%)	30 (40%)	
Mean ± SD	39.5 ± 3.63	38.88 ± 3.23	39.29 ± 3.49	0.472^‡^
Median (25th-75th percentile)	39 (37-42.75)	38 (37-41)	39 (37-42)	
Range	32-45	34-45	32-45	
Height (cm)				
Mean ± SD	154.36 ± 4.69	154.4 ± 4.16	154.37 ± 4.49	0.971^‡^
Median (25^th ^to 75th percentile)	154 (151-158)	153 (152-158)	154 (151-158)	
Range	147-164	147-162	147-164	
Weight (kg)				
Mean ± SD	67.68 ± 4.67	68 ± 5.2	67.79 ± 4.82	0.789^‡^
Median (25^th^ to 75th percentile)	67.5 (65-71)	67 (65-70)	67 (65-71)	
Range	58-78	60-79	58-79	
Hemoglobin (g/dL)				
Mean ± SD	8.59 ± 0.6	10.8 ± 0.59	9.32 ± 1.21	<.0001^‡^
Median (25^th^ to 75th percentile)	8.5 (8.2-8.9)	10.8 (10.4-11.2)	8.9 (8.35-10.45)	
Range	7.8-11	9.8-11.8	7.8-11.8	

Similarly, significant associations were observed in uterine artery Doppler indices (Table 2), specifically peak systolic velocity (PSV) and end-diastolic velocity (EDV), which had higher values in Group A as compared to Group B. However, no significant association was observed for the RI and PI.

**Table 2:** Association of uterine artery Doppler Indices with heavy menstrual bleeding ‡ Independent t-test, § Mann Whitney test HMB: heavy menstrual bleeding

Uterine artery Doppler indices	Participants with HMB (n=50)	Participants without HMB (n=25)	Total (n=75)	P-value
Peak systolic velocity (cm/s)				
Mean ± SD	53.38 ± 12.8	33.06 ± 4.54	46.61 ± 14.43	<.0001^‡^
Median (25th-75th percentile)	50.51 (43.61-56.76)	34.07 (29.32-36.4)	43.44 (36.5-52.195)	
Range	40.59-89.9	23.65-38.78	23.65-89.9	
End-diastolic velocity (cm/s)				
Mean ± SD	12.34 ± 7.07	9.33 ± 7.03	11.34 ± 7.15	0.028^§^
Median (25th-75th percentile)	9.84 (6.825-17.758)	6.36 (4.71-10.97)	8.5 (5.78-17.03)	
Range	2.8-29.8	2.5-26.9	2.5-29.8	
Resistive index				
Mean ± SD	0.89 ± 0.39	0.74 ± 0.23	0.84 ± 0.35	0.087^‡^
Median (25th-75th percentile)	0.78 (0.678-0.878)	0.79 (0.72-0.86)	0.78 (0.685-0.87)	
Range	0.51-2	0.26-1.21	0.26-2	
Pulsatility index				
Mean ± SD	2.6 ± 2.97	1.87 ± 1.18	2.36 ± 2.53	0.653^§^
Median (25th-75th percentile)	1.6 (1.16-2.398)	1.61 (1.31-2.05)	1.61 (1.16-2.385)	
Range	0.54-13.79	0.47-5.01	0.47-13.79	

Likewise, significant associations were observed in Doppler indices of the dominant feeding vessel of fibroid between Group A and Group B. PSV and EDV had significantly higher values in Group A as compared to Group B, while Resistive Index (RI) and Pulsatility Index (PI) had higher values in Group B as compared to Group A (Table 3)

**Table 3:** Association of Doppler Indices in the dominant feeding vessel of fibroid with heavy menstrual bleeding ‡ Independent t-test, § Mann Whitney test HMB: heavy menstrual bleeding

Doppler indices in the dominant feeding vessel of fibroid	Participants with HMB (n=50)	Participants without HMB (n=25)	Total (n=75)	P value
Peak systolic velocity (cm/s)				
Mean ± SD	26.42 ± 4.57	14.56 ± 3.17	22.46 ± 6.98	<.0001^‡^
Median (25th-75th percentile)	25.55 (22.752-28.877)	14.1 (12.5-16.4)	22.5 (16.6-27.92)	
Range	20.16-38.06	7.55-19.5	7.55-38.06	
End-diastolic velocity (cm/s)				
Mean ± SD	8.68 ± 4.35	5.14 ± 2.75	7.5 ± 4.22	<.0001^§^
Median (25th-75th percentile)	6.76 (6.152-8.74)	3.99 (3.2-5.95)	6.29 (5.155-8.655)	
Range	5.1-21.3	1.6-12.3	1.6-21.3	
Resistive index				
Mean ± SD	0.44 ± 0.14	0.85 ± 0.91	0.58 ± 0.57	0.034^‡^
Median (25th-75th percentile)	0.44 (0.382-0.49)	0.69 (0.61-0.75)	0.48 (0.395-0.655)	
Range	0.09-0.81	0.22-5.1	0.09-5.1	
Pulsatility index				
Mean ± SD	0.7 ± 0.32	1.14 ± 0.66	0.84 ± 0.51	<.0001^§^
Median (25th-75th percentile)	0.65 (0.572-0.765)	0.98 (0.76-1.48)	0.68 (0.59-0.92)	
Range	0.17-2.12	0.26-3.44	0.17-3.44	

**Figure 2:**
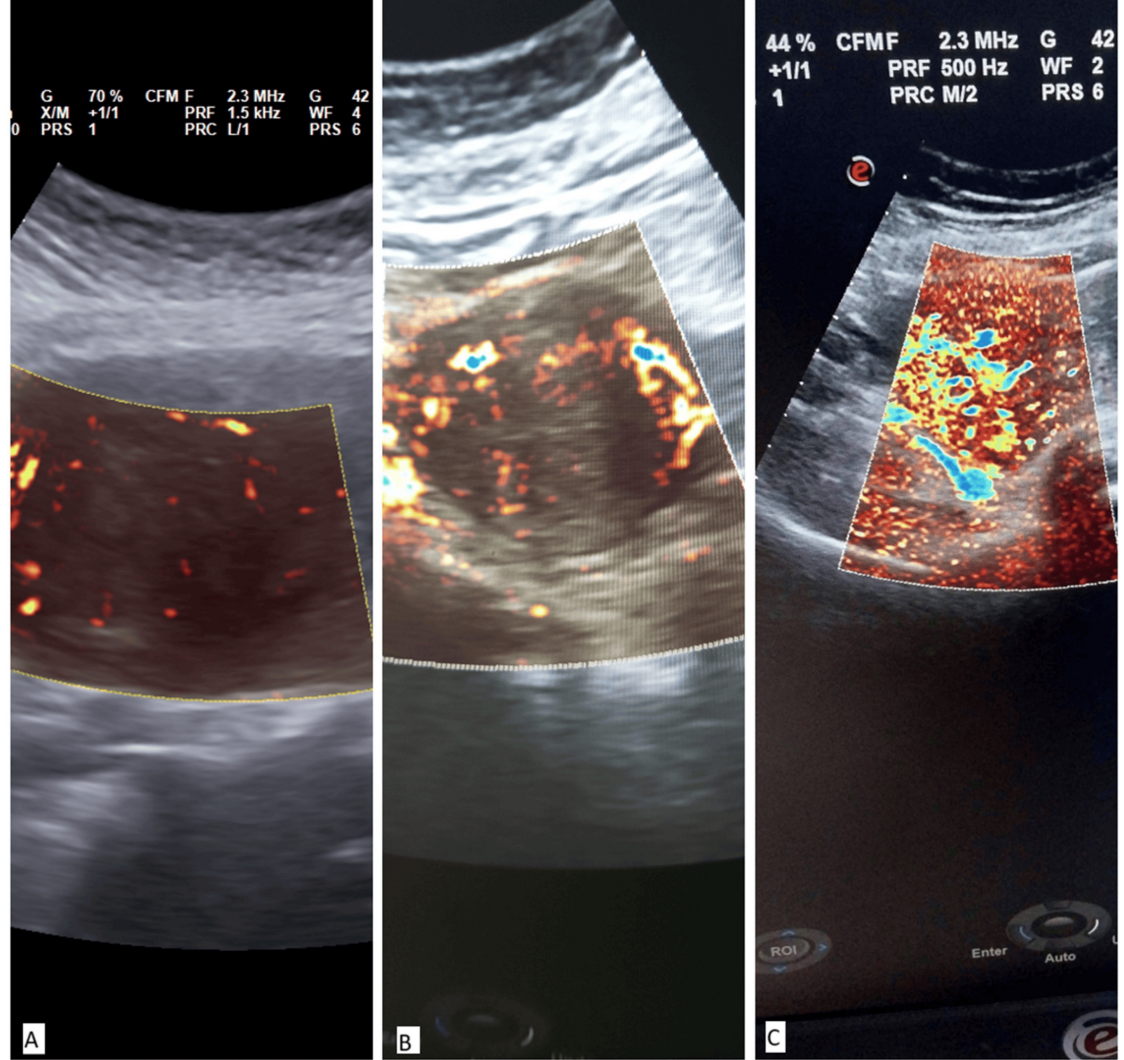
Grade of vascularity of dominant fibroid on SMI (A) Grade 1 (minimal vascularity); (B) Grade 2 (few small vessels and/or a main vessel seen); (C) Grade 3 (4 or more vessels visualized) SMI: superb microvascular imaging

**Figure 3:**
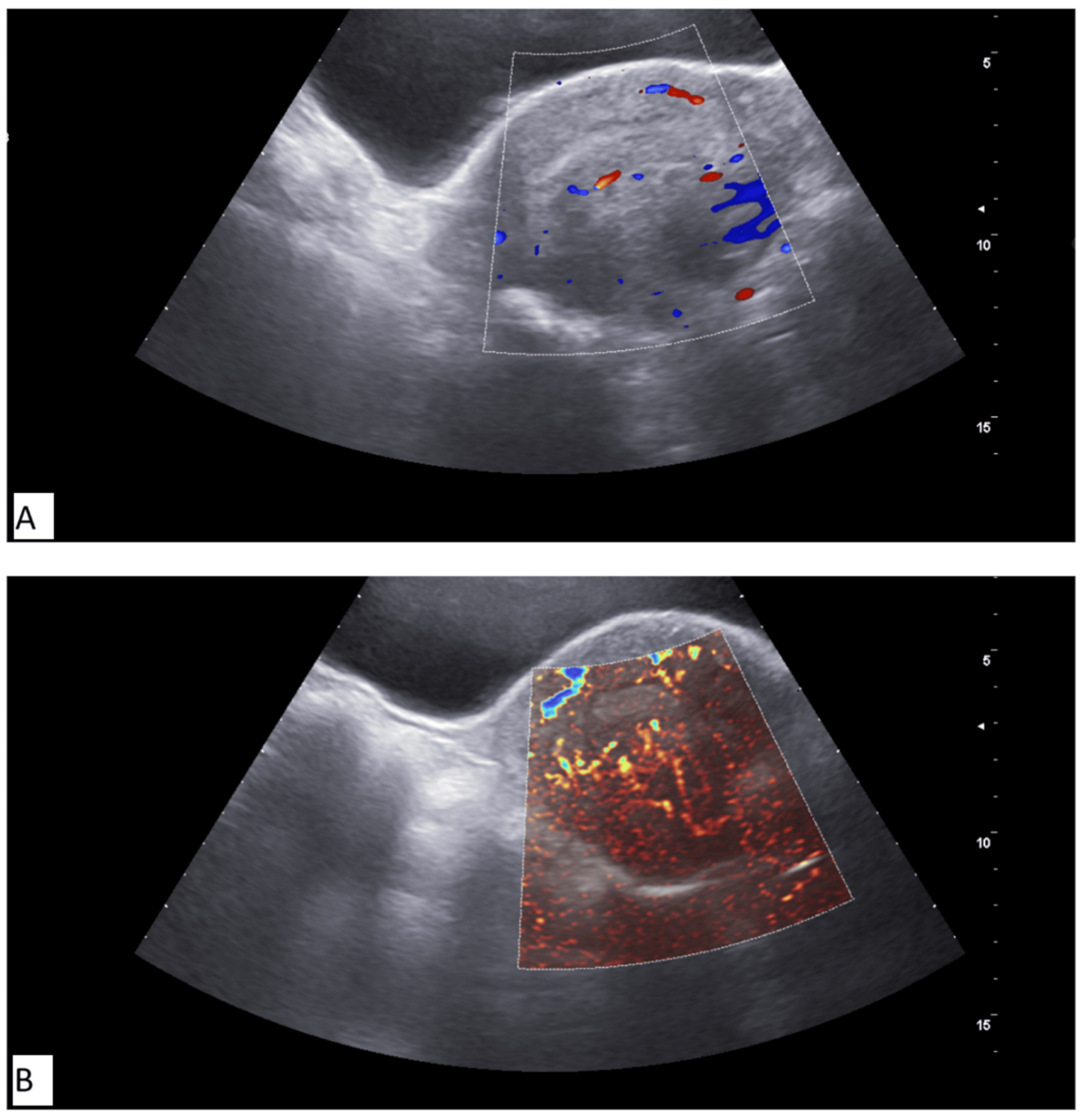
Vascularity of dominant fibroid on color Doppler and SMI (A) Color Doppler showing grade 1 vascularity; (B) SMI showing grade 2 vascularity SMI: superb microvascular imaging

SMI was used to study vascularity in the dominant fibroid, which showed better vascular architecture than CDUS (Figures 2, 3). The proportion of women exhibiting both central and peripheral vascularity in fibroids was significantly higher in Group A whereas participants without HMB (Group B) had mostly peripheral vascularity in fibroids. Also, Group A showed significantly higher proportions of those showing grade 2 and grade 3 vascularity in fibroids, while participants without HMB (Group B) had mostly grade 1 vascularity (Table 4).

**Table 4:** Association of vascular pattern and grade of vascularity in fibroid with heavy menstrual bleeding * Fisher's exact test HMB: heavy menstrual bleeding

Vascular pattern/ Grade of vascularity	Participants with HMB (n=50), n (%)	Participants without HMB (n=25), n (%)	Total (n=75), n (%)	P value
Vascular Pattern				
Peripheral	14 (28%)	24 (96%)	38 (50.67%)	<.0001^*^
Both{Central + Peripheral}	36 (72%)	1 (4%)	37 (49.33%)	
Total	50	25	75	
Grade of vascularity				
Grade 1	2 (4%)	25 (100%)	27 (36%)	<.0001^*^
Grade 2	43 (86%)	0	43 (57.33%)	
Grade 3	5 (10%)	0	5 (6.66%)	
Total	50	25	75	

Table 5 depicts the association of uterine volume and size of fibroid with HMB. Group A had significantly higher uterine volume and size of fibroid.

**Table 5:** Association of uterine volume and fibroid size with heavy menstrual bleeding ‡ Independent t-test HMB: heavy menstrual bleeding

Uterine volume/ Size of fibroid	Participants with HMB (n=50)	Participants without HMB(n=25)	Total (n=75)	P value
Uterine volume (cc)				
Mean ± SD	166.82 ± 34.72	130.64 ± 17.62	154.76 ± 34.55	<.0001^‡^
Median (25th-75th percentile)	155.5 (143-186.75)	128 (118-146)	148 (132-162.5)	
Range	115-246	102-159	102-246	
Size of fibroid (cm)				
Mean ± SD	4.81 ± 1.42	3.32 ± 0.81	4.31 ± 1.43	
Median (25th-75th percentile)	4.5 (3.925-5.475)	3.5 (2.6-4)	4.2 (3.45-4.75)	<.0001^‡^
Range	2.4-7.9	2.1-5	2.1-7.9	

In Group A, a significant moderately positive correlation and a significant weak positive correlation were seen between uterine volume with PSV and RI respectively. While non-significant very weak positive correlation was seen between uterine volume with EDV and PI. Similarly, in Group B, a non-significant very weak positive correlation was seen between uterine volume with RI, PI, and a non-significant weak positive correlation with PSV; while a non-significant weak negative correlation with EDV (Table 6).

**Table 6:** Correlation of uterine volume with Doppler indices (PSV, EDV, RI, and PI) in women with and without HMB ** Pearson correlation coefficient, ¶ Spearman rank correlation coefficient PSV: peak systolic velocity; EDV: end-diastolic velocity; RI: resistive index; PI: pulsatility index; HMB: heavy menstrual bleeding

Variables	PSV	EDV	RI	PI
Uterine volume in women with HMB (Group A)				
Correlation coefficient	0.519	0.118	0.365	0.194
P value	0.0001^**^	0.415^¶^	0.009^**^	0.177^¶^
Uterine volume in women without HMB (Group B)				
Correlation coefficient	0.273	-0.112	0.171	0.187
P value	0.186^**^	0.593^¶^	0.413^**^	0.370^¶^

## Discussion

Uterine fibroids are the most common benign tumors of the uterus, mostly seen in the reproductive age group. They are usually asymptomatic but in some cases may present with HMB, which can be detrimental to the overall health of women. Many need to undergo surgical procedures like hysterectomy or myomectomy, while they can be managed by more conservative procedures, like UAE and HIFU. Several previous studies have correlated uterine artery Doppler findings with the outcome of uterine artery embolization. Also, assessment of peri-fibroid and intra-fibroid Doppler indices have been found clinically useful in pre-UAE procedures because the overall tumor vascularity is said to be predictive of the therapeutic outcome of UAE. Hyper-vascular fibroids show more reduction in size post-UAE than iso-vascular and hypo-vascular fibroids. In the current study, we tried to find the association of HMB with the vascularity of fibroid and uterine artery Doppler findings. In the current study, we studied Doppler indices of the uterine artery and the dominant vessel of fibroid in both groups (women with or without HMB). We also used a newer modality, SMI, for determining the vascular pattern and grading of blood flow in the fibroid and its correlation between the two groups.

Women with intramural uterine fibroid (pre-diagnosed on ultrasound) who met the inclusion criteria were grouped into two groups: group A (with HMB) and group B (without HMB). The two groups were compared for age, height, weight, Hb, uterine volume, fibroid size, doppler indices of dominant vessel of fibroid & uterine artery; and vascular pattern and grading in fibroid. The mean age of group A was 39.5 ± 3.63 years and that of group B was 38.88 ± 3.23 years. The association between age and the presence of HMB was not statistically significant, with a p-value of 0.472. The majority of women belonged to the age group of 36-40 years because fibroid is most common in the reproductive age group. In a similar study conducted by Saad et al., there was no significant difference between women with fibroid uterus with or without abnormal uterine bleeding regarding age, as their ages were 42.21 ± 1.71 and 42.47 ± 1.73 years, respectively [16]. In a study by Idowu et al., the mean age of women with fibroid was 37.9 ± 7.4 years [17]. Similarly, there was no significant difference between anthropometric parameters such as height and weight in the two groups (p-value=0.971 and 0.789, respectively). There was a notable difference in Hb levels among the two groups; mean Hb levels in patients without HMB were 10.8 ± 0.59, significantly higher compared to patients with HMB, where it was 8.59 ± 0.6 (p-value < 0.0001). This finding is similar to Saad et al. where mean Hb was 8.97 + 0.96 in women with bleeding, significantly higher as compared to women without bleeding (11.15 + 1.13) with p-value < 0.001 [16]. This difference is expected due to the presence of HMB in women in Group B.

A significant association was observed between uterine artery Doppler indices, specifically, PSV and EDV, and HMB. For PSV in the uterine artery Doppler, the mean ± SD in patients with HMB was 53.38 ± 12.8 cm/s, significantly higher compared to patients without HMB, where it measured 33.06 ± 4.54 cm/s (p-value < 0.0001). Regarding EDV in the uterine artery Doppler, the median (25th-75th percentile) in patients with HMB was 9.84 (6.825-17.758) cm/s, significantly higher compared to patients without HMB, where it was 6.36 (4.71-10.97) cm/s (p-value = 0.028). However, no significant association was found in the RI, with a p-value of 0.087. The mean ± SD of RI in patients with HMB was 0.89 ± 0.39, and in patients without HMB was 0.74 ± 0.23, indicating no significant difference between the groups. Similarly, no significant association was found in the PI, with a p-value of 0.653. The median (25th-75th percentile) of PI in patients with HMB was 1.6 (1.16-2.398), and in patients without HMB was 1.61 (1.31-2.05), showing no significant difference between the groups. In the study by Saad et al., women with bleeding had significantly higher mean values for PSV and EDV and lower RI and PI values as compared to women without HMB [16]. The mean PSV was 130.34 ± 10.90 in women with HMB, while the mean PSV was 56.76 ± 13.1 in women without HMB, with a p-value <0.001. The mean EDV was 40.58 ± 5.15, significantly higher than women without HMB (38.00 ± 8.69). The mean RI was 0.52 ± 0.02 in women with HMB, significantly lower than women without bleeding (0.61 ± 0.09). The mean PI was 0.71 ± 0.05 in women with HMB, significantly lower than women without HMB (0.93 ± 0.34), with a p-value<0.001. Ait‑Allah et al., in a similar study in Egypt in 2021, found that the uterine blood flow was significantly higher and the Doppler indices were significantly lower in women with menorrhagia. Vascular index (VI) which denotes baseline fibroid vascularization was significantly associated with Pictorial Blood Loss Assessment Chart (PBAC) score; a 1% higher VI at baseline led to an 11-point increase in PBAC score over time (RC 10.99, p=0.05, 95%CI -0.15 - 22.12) [18]. 

Significant associations were observed in Doppler indices in the dominant feeding vessel of fibroids between women with and without HMB. For PSV in the dominant feeding vessel of fibroids, the mean ± SD in women with HMB was 26.42 ± 4.57, significantly higher compared to women without HMB (14.56 ± 3.17) (p-value < 0.0001). Regarding EDV, the median (25th-75th percentile) in women with HMB was 6.76 (6.152-8.74), significantly higher compared to women without HMB (3.99 (3.2-5.95)) (p-value < 0.0001). Similarly, for the RI in the dominant feeding vessel of fibroids, the mean ± SD in women without HMB was 0.85 ± 0.91, significantly higher than in women with HMB (0.44 ± 0.14) (p-value = 0.034). Lastly, for the PI in the dominant feeding vessel of fibroids, the median (25th-75th percentile) in women without HMB was 0.98 (0.76-1.48), significantly higher compared to women with HMB (0.65 (0.572-0.765)) (p-value < 0.0001). This finding is corroborated in the study by Keizer-et al., where the degree of fibroid vascularization (measured by 3D PDUS) is related to deteriorating symptoms over time (during 12 months follow-up) [12]. This is in contrast to the study by Idowu et al, where no statistically significant difference was observed between the degree of vascularity of the dominant fibroid nodules in symptomatic women and those of asymptomatic ones [17]. 

To study the vascular pattern and grade of vascularity in fibroid, we have used SMI, a new and advanced Doppler ultrasound technique designed to detect slow-velocity blood flow in small-caliber micro-vessels. Microvascular imaging provided a more detailed impression of the vascular architecture than PDUS [19]. The proportion of patients exhibiting a vascular pattern characterized by both central and peripheral features was significantly higher in those with HMB compared to those without HMB, with percentages of 72% and 4%, respectively. Conversely, the proportion of patients with only peripheral vascularization was significantly lower in individuals with HMB compared to those without HMB, with percentages of 28% and 96%, respectively (p-value < 0.0001). In the literature reviewed, no study was found to correlate this finding.

The proportion of patients with different grades of vascularity exhibited significant differences between those with HMB and those without HMB. Patients with HMB showed a significantly higher proportion of both grade 2 vascularity and grade 3 vascularity (76% and 20% respectively) and only 4% had grade 1 vascularity. While women without HMB exhibited only grade 1 vascularity. These differences were statistically significant, with a p-value of <0.0001. To the best of our knowledge, no study has been conducted to correlate the grade of vascularity in fibroid with HMB.

Although we restricted the fibroid size to 2-8 cm and a maximum of two in number, even within this size restriction, the mean size of fibroids among patients with HMB was 4.81 ± 1.42, which was significantly higher compared to patients without HMB, where it measured 3.32 ± 0.81 (p-value < 0.0001). The mean uterine volume among patients with HMB was 166.82 ± 34.72, significantly higher compared to patients without HMB, where it was 130.64 ± 17.62 (p-value < 0.0001). This indicates a significant difference in uterine volume between the two groups. In the study done by Saad et al, the mean uterine volume among women with bleeding was 215.15 ± 8.16, significantly higher as compared to women without bleeding (174.20 ± 14.75) with p-value < 0.0001 [16]. Ait‑Allah et al. found that the uterine volume was significantly higher in cases of menorrhagia, which may be due to multiparty and the associated increase in the uterine volume and increased vascularity in some cases and the presence of some pathology (leiomyoma in some and adenomyosis in others) [18].

In women with HMB, a significant weak positive correlation was seen between uterine volume and RI with a correlation coefficient of 0.365. A significantly moderately positive correlation was seen between uterine volume and PSV with a correlation coefficient of 0.519. A non-significant very weak positive correlation was seen between uterine volume with EDV, and PI with a correlation coefficient of 0.118 and 0.194 respectively. In women without HMB, a non-significant very weak positive correlation was seen between uterine volume with RI, and PI with a correlation coefficient of 0.171 and 0.187, respectively. A non-significant weak positive correlation was seen between uterine volume and PSV with a correlation coefficient of 0.273. A non-significant very weak negative correlation was seen between uterine volume and EDV with a correlation coefficient of -0.112. In the study done by Saad et al., there was no significant correlation between uterine volume and uterine artery Doppler indices in women without bleeding and those with bleeding [16]. Ait‑Allah et al. found a strong positive correlation between the uterine volume and Doppler blood flow and a strong negative correlation with the Doppler indices of the uterine arteries in cases of menorrhagia, whereas only a positive correlation with PI in regularly menstruating women [18].

Limitations of the study

The study had some limitations. Firstly, the ultrasound finding of fibroid was not confirmed with histopathological examination. Leiomyosarcomas and focal adenomyoma may rarely be confused with leiomyoma. Secondly, the ultrasound was performed by two equally qualified radiologists, but still, it might have led to observer bias. Thirdly, although we had limited our study to intramural fibroids of size 2-8 cm, there was variation in the size of fibroids in the two groups which may have influenced HMB. Lastly, the sample size was small and a larger study may be needed to validate our findings.

## Conclusions

Doppler indices in the dominant feeding vessel of fibroid show a positive correlation with HMB. The percentage of women exhibiting both central and peripheral vascularity on superb microvascular imaging was much higher in group A. Individuals in Group A showed a significantly higher proportion of grade 2 and grade 3 vascularity. Conversely, in Group B, all the individuals showed grade 1 vascularity. Fibroid vascularization assessed with 3D PDUS has been shown in earlier studies to be a predictor for fibroid-related symptoms, quality of life, and uterine fibroid growth. Vascularization of fibroid is also directly related to response with medical management like GnRH agonists and letrozole and uterus preserving minimally invasive procedures like UAE.

Most women with symptomatic fibroids are of childbearing age and fertility preservation is at times their prime concern. Fibroid vascularization assessed with 3D PDUS and the new advanced SMI tool may prove to be useful in the prediction of response to the various non-surgical management options of uterine leiomyoma and thus aid in counseling and appropriate patient selection.
